# Broadband, Compact, and Training-Free Optical Processors for Parallel Image Classification

**DOI:** 10.1021/acsnano.6c03355

**Published:** 2026-06-19

**Authors:** Sander J. W. Vonk, Boris de Jong, Yannik M. Glauser, David B. Seda, Matthieu F. Bidaut, Benjamin Savinson, Hannah Niese, David J. Norris

**Affiliations:** Optical Materials Engineering Laboratory, Department of Mechanical and Process Engineering, ETH Zurich, Zurich 8092, Switzerland

**Keywords:** optical computing, Fourier surfaces, image classification, nanofabrication, machine learning

## Abstract

As artificial intelligence becomes increasingly prevalent, the demand for faster and more energy-efficient computing approaches grows. While optical computing offers intrinsic advantages in bandwidth and power consumption, existing implementations remain bulky, wavelength-specific, and dependent on complex training procedures, limiting scalability and parallel operation. In this work, we demonstrate a compact, training-free optical processor based on wavy diffractive structures, known as Fourier surfaces, for parallel image classification. Our device achieves all-optical classification accuracies of up to 76% for digits and 59% for fashion items within a 40 × 40 μm^2^ footprint. A linear matrix operation on the optical outputs boosts the accuracies to 84% and 66%. The diffractive layer inherently separates incident wavelengths into distinct output directions, enabling broadband operation and allowing multiple colors to function as independent computation channels. As a result, this passive system supports up to 6 simultaneous computations. Theoretical inverse-designed extensions show that all-optical classification accuracies up to 94% are achievable within the same physical framework, and on-chip photonic integration could further enhance performance by enabling nonlinear transformations.

## Graphical abstract

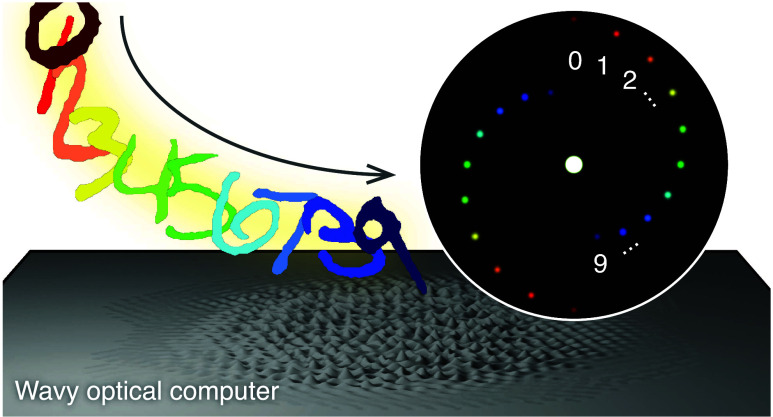

## Introduction

As artificial intelligence (AI) systems continue to grow in complexity and size, their power demands are emerging as a critical bottleneck.^1^ Energy consumption of state-of-the-art AI models is already straining data infrastructure and depleting resources. Alternative computing architectures are therefore needed that can deliver high performance at drastically reduced energy costs.

Optical computing offers a promising solution due to the inherently high bandwidth and low-loss nature of light.^2,3^ Optical systems can perform computations with far greater energy efficiency than electronic processors,^2^ with recent demonstrations even approaching subphoton energy usage per operation.^4^ Research in optical computing has therefore accelerated, spanning diffractive and scattering-based approaches in free-space^5–15^ or on-chip^16–21^ aimed at overcoming the limitations of electronics.

However, many of these implementations remain bulky and rely on large optical components such as spatial-light modulators or extended cascaded layers. In many cases, this large footprint arises from efforts to achieve performance on par with digital neural networks. While such approaches demonstrate the potential of optical systems for high-accuracy inference, they often come at the cost of reduced scalability. Furthermore, the design and training of optical networks pose significant challenges,^22,23^ as optimizing the device parameters for a given computational task often requires large training data sets and iterative optimization. Achieving broadband operation adds an additional layer of difficulty, since the performance of diffractive and scattering-based systems is often highly wavelength-dependent and typically requires deep-learning-assisted design to extend functionality across multiple wavelengths.^24,25^ These challenges have motivated the search for compact, easily trainable optical architectures capable of achieving high computational density and broadband operation.

Here, we design, fabricate, and benchmark an optical processor based on wavy diffractive structures, known as Fourier surfaces, for parallel image classification.^26^ This wavy approach is enabled by the continuous height control enabled by thermal scanning probe lithography (TSPL), which allows the fabrication of smoothly varying, wavy diffractive surfaces rather than discretized or binary patterns. As such, the device operates without iterative training: the surface height profile is determined from training data sets in a single analytical step, without gradient-based optimization or backpropagation. By operating directly on optical image inputs, the processor avoids intermediate electro–optical conversion, reducing latency and energy consumption. Moreover, rather than relying on deep architectures to maximize classification accuracy, our approach focuses on compact and broadband optical processors for scalable and parallel photonic computing.

Each output channel corresponds to a characteristic feature pattern derived from the image data set, enabling intensity-based classification. Because the diffracted outputs for a single color are directed to distinct polar angles, the architecture is theoretically broadband, allowing different wavelengths to function as independent computational channels. Theory shows that up to 20 wavelengths can be resolved simultaneously, and experiments confirm robust all-optical inference for multiplexing factors up to six. Overall, our single-layer design achieves all-optical classification accuracies of up to 76% for handwritten digits and 59% for fashion items within a compact 40 × 40 μm^2^ footprint, while enabling multiwavelength parallel processing—a combination that highlights its potential for scalable, low-power, and high-throughput photonic computing. In addition, inverse-designed extensions suggest further improvements in all-optical classification performance within the same framework (86–94%), while hybrid free-space/on-chip implementations could yield even more expressive optical processing.

## Results and Discussion

### Training-Free Optical Classification in Fourier Space

Figure 1a schematically shows the working principle of the single-layer optical processor. We project an optical input, in this example an amplitude image of a zero from the digit MNIST data set^27^, onto the wavy silver diffractive surface. The diffractive surface introduces local optical path length differences, imprinting a spatially varying phase onto the incident amplitude input. This amplitude and phase profile diffracts into predefined directions that are collected by our microscope objective. We image the back focal plane (Fourier space) of our microscope objective to directly map the diffraction angles of the optical processor. A high optical intensity in the designated output port leads to a successful classification of the input image.

**Figure 1:**
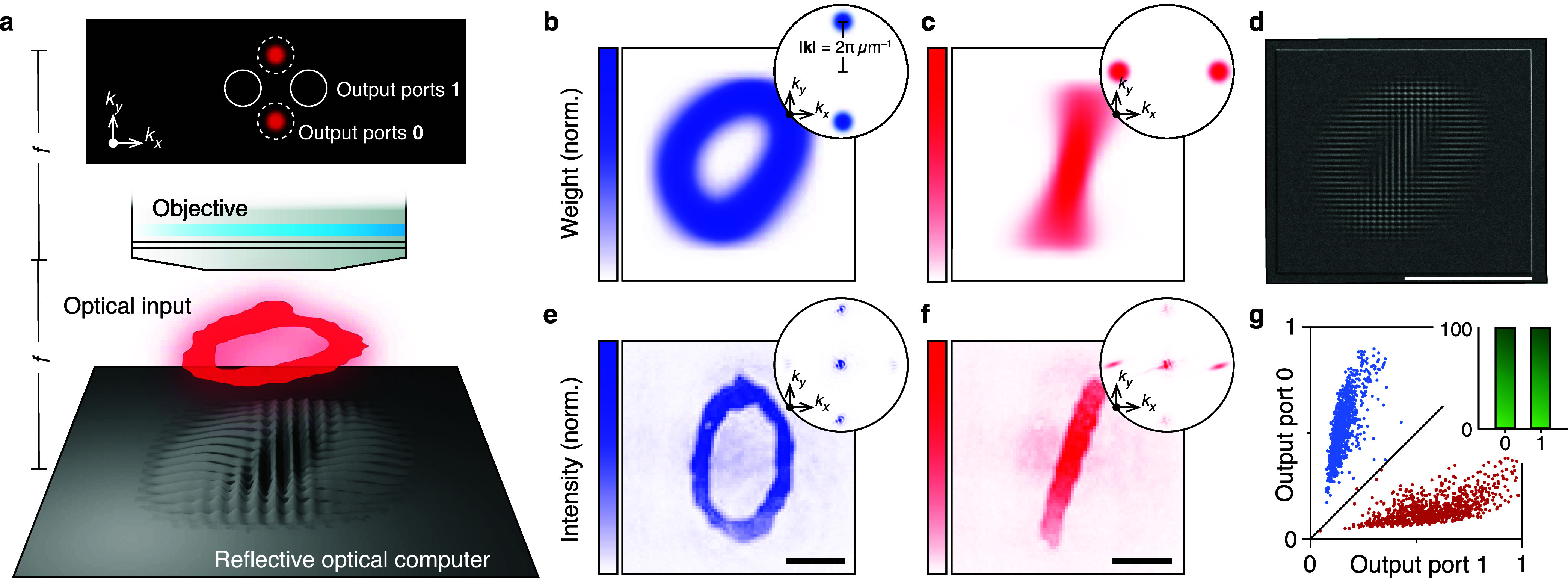
Training-free optical classification. (a) Schematic of the working principle of the compact reflective optical processor. Optical input images (encoded in amplitude) from the digit MNIST test set are projected onto the wavy diffractive surface. The resulting diffraction propagates to the back focal plane (Fourier space) of the microscope objective. Comparing the integrated intensities in all output ports enables classification of the optical input. (b,c) Average spatial distributions of the digit classes zero and one, used as spatial weights for constructing class-specific sinusoidal gratings with 1 μm pitch. Inset: Fourier spectrum of sinusoidal gratings in (b) *y*-direction and (c) *x*-direction. (d) Scanning electron micrograph (SEM; 30° tilt) of the fabricated 40 × 40 μm^2^, 150 nm-deep computing layer in silver (Ag). The scalebar is 20 μm. (e,f) Reflected real-space images from flat silver of a projected (e) zero and (f) one. Scale bars are 10 μm. Insets: measured Fourier-space maps showing dominant diffraction into the class-specific predefined output directions. (g) Measured normalized output intensities for all 2115 test images (980 zeros and 1135 ones). A correct classification occurs when the designated output channel has the highest intensity. Here, we achieve a classification accuracy of 99.9%. Inset: histogram of classification accuracy per input class.

The design of the optical processor is entirely training-free, and its wavy structure is built up from diffractive grating structures that serve as class-specific spatial templates. As a simple example, Figure 1 shows the design for a binary classifier for input images of zeros and ones. For both classes in this data set, we compute the average spatial distribution (Figure 1b,c; all zeros and ones in the training set), which acts as a spatial weighting of sinusoidal gratings with a pitch *p* = 1 μm and a specific diffraction direction. This value of 1 μm is chosen as a trade-off between angular separation and collection efficiency, ensuring that visible wavelengths diffract within the numerical aperture of our microscope objective, while maintaining sufficient separation from the specular reflection. The grating diffracts into the *x*-direction for the one and the *y*-direction for the zero (Figure 1b,c; insets show the two-dimensional Fourier spectrum). Superposition of these weighted gratings forms the design for a single diffractive layer that maps distinct amplitude images to specific angularly separated diffraction orders, or output ports.

For the fabrication, the wavy design (Figure S1 in the Supporting Information) is first patterned into a thermoresponsive polymer using TSPL.^26,28^ Here, the maximum depth of the structure is fixed to 150 nm. After patterning, silver is evaporated onto the polymer^29^ and finally, the optical processor is obtained through template stripping.^30^ Figure 1d shows a scanning electron micrograph (SEM) of the optical processor.

For binary inference, we illuminate the device with input test images at a wavelength λ = 650 nm using a phase-only spatial-light modulator (SLM). By placing the SLM in the illumination path between crossed polarizers, we achieve images at the device via amplitude modulation (see Figure S2 in Supporting Information for the optical setup). Figure 1e,f shows examples of recorded real-space images of a projected zero and a projected one reflected from flat silver. For this, the 28 × 28 pixel images are digitally upscaled to cover 960 × 960 SLM pixels (7.7 × 7.7 mm^2^), such that the image of the SLM plane matches the size of the reflective layer (40 × 40 μm^2^) after propagating through our optical setup (see Methods). The spatial overlap of the input image with the corresponding grating template maximizes the diffracted intensity in the correct output direction (Figure 1e,f; insets for corresponding measurements of Fourier space), enabling optical classification. The bright spot in the center of Fourier space is the specular reflection of the input image. For each measurement *n*, we integrate the intensity over both the +1 and −1 diffraction orders for each output channel to obtain the raw optical output ***x*** ^(*n*)^. For this, we integrate over a circular region in Fourier space with a radius of *k*_∥_/*k*_0_ = 0.03 (with *k*_0_ = 2π/*λ* and *k*_∥_ = 0.28 μm^–1^ at 650 nm), corresponding to approximately 18 pixels. Then, we renormalize by the average intensity per output channel over all measurements ***x*** *®* to obtain the normalized optical output ${\mathrm{x̃}}^{\left(n\right)}=x^{\left(n\right)}⌀\mathrm{x̅}$ , with ⌀ representing element-wise division. This renormalization compensates for differences in the average spatial extent of images across input classes; for example, zeros occupy a larger area on average than ones. Theoretically, light can also be diffracted by higher-order diffraction processes, but these have low efficiencies for our shallow diffractive surfaces (see Figure S3 in the Supporting Information). Figure 1g shows the output intensities in the two output channels for all zeros (blue dots, 980 inputs) and ones (red dots, 1135 inputs) from the digit MNIST test set. A successful classification has the highest intensity in the designated output port, so *y* > *x* (Figure 1g, black line gives *y* = *x*) for a zero and *y* < *x* for a one. We achieve classification accuracies of 99.9% and 99.8% for zeros and ones, respectively (Figure 1g, inset).

### Full-Digit Classification

Building upon the binary optical classifier, we extend the training-free concept to full-digit classification by simply encoding ten distinct sinusoidal gratings within a single reflective silver layer. Each grating corresponds to a digit class (0–9) and is oriented at a specific azimuthal angle φ. The angles are spaced by Δφ = π/10, such that each class diffracts light into a distinct output direction and the optical outputs are evenly distributed in Fourier space. The superposition of these gratings forms a compact diffractive surface that again directs optical inputs to class-specific output ports based on their spatial overlap with the corresponding spatial template. Figure 2a shows an SEM of the fabricated optical processor in silver. Visually, overlapping features from different digit classes superpose their gratings, producing regions of enhanced structural symmetry reminiscent of quasicrystalline order.^26^ The optical output in Fourier space (Figure 2b) for an input image of a zero, clearly shows that the diffracted intensity is maximal into the two output directions assigned to the zero class, consistent with a successful classification. For visualization, the plotted intensity is clipped to 0.1 *I*/*I*_max_, as the fixed 150 nm depth reduces the diffraction efficiency of each individual sinusoid in the full ten-class device.

**Figure 2:**
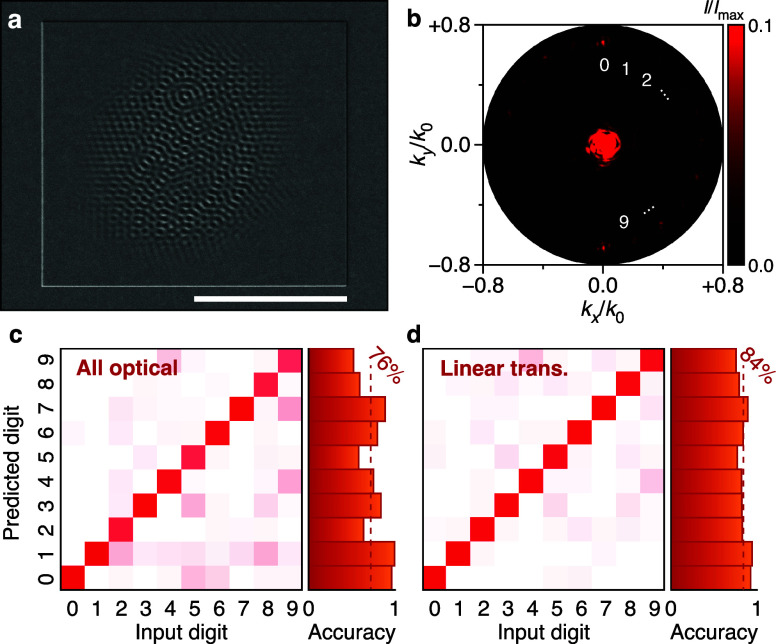
Full-digit classification. (a) SEM (30° tilt) of the fabricated 40 × 40 μm^2^, 150 nm-deep diffractive layer, comprising ten class-specific sinusoidal gratings oriented at azimuthal angles incremented by π/10. The scalebar is 20 μm. (b) Measured Fourier-space map for an input image of a zero, showing dominant diffraction into the two output directions associated with the zero class. The intensity is clipped to 0.1 *I*/*I*_max_ for visibility of the diffraction orders with respect to bright specular reflection. (c) Confusion matrix for all-optical inference across the 10,000 test images. Inset: single-digit accuracies (histogram) and average accuracy η = 76% (dashed line). (d) Confusion matrix and single-digit accuracies after applying an optimized 10 × 10 linear calibration matrix **M** to the optical outputs. The classification accuracy increases to η = 84%.

To quantitatively validate the performance of the full-digit classifier, we benchmark it using all *N* = 10,000 test images from the digit MNIST data set. For input *n*, the optical response vector ${\mathrm{x̃}}^{\left(n\right)}$ is obtained using the same readout/normalization procedure as for the binary classification (Figure 1), but now with 10 output classes with labels *t* ^(*n*)^ ∈ [0, 9]. The predicted class label is then determined as ${\mathrm{t̂}}^{\left(n\right)}=\arg\;\max{\mathrm{x̃}}^{\left(n\right)}$ , where arg max(·) returns the element (or output port) of ${\mathrm{x̃}}^{\left(n\right)}$ with the highest intensity. From these measurements, we construct the confusion matrix

1 $$P_{i,j}=\frac{\sum_{n=1}^{N}\delta_{t^{\left(n\right)},i}\delta_{{\mathrm{t̂}}^{\left(n\right)},j}}{\sum_{n=1}^{N}\delta_{t^{\left(n\right)},i}}$$

which gives the fraction of the inputs of true class *i* being interpreted as class *j* (Figure 2c). Here, δ_*i*,*j*_ denotes the Kronecker delta, equal to 1 when *i* = *j* and 0 otherwise. The histogram (inset) shows the single-digit classification accuracy *P*_*i*,*i*_. The overall classification accuracy η is obtained from the diagonal elements of the confusion matrix as

2 $$\eta =\frac{1}{N}\sum_{n=1}^{N}\delta_{t^{\left(n\right)},{\mathrm{t̂}}^{\left(n\right)}}$$

yielding an accuracy of η = 76% for full all-optical classification across ten digit classes. The influence of lateral input misalignment on classification accuracy is analyzed in Figure S4 in the Supporting Information, showing performance degradation with horizontal (Δ*x*) and vertical (Δ*y*) shifts, dropping below 60% for |Δ*x*| > 1 μm and |Δ*y*| > 2 μm.

To boost the classification accuracy with minimal additional electronic computational effort, we introduce a simple linear correction step applied to the optical outputs ${\mathrm{x̃}}^{\left(n\right)}$ (Figure 2d). More specifically, we train a 10 × 10 matrix **M** that performs a linear transformation on the measured intensity vectors

3 $${ỹ}^{\left(n\right)}=M{\mathrm{x̃}}^{\left(n\right)}$$

such that the transformed outputs ${ỹ}^{\left(n\right)}$ maximize the overall classification accuracy. This postprocessing step can be interpreted as a calibration of the optical response, compensating for likely channel crosstalk between sets of output ports. Figure 2d presents the resulting confusion matrix and single-digit accuracy histogram obtained using a matrix **M** trained on the full set of measured optical responses. The classification accuracy increases from 76% to 84%, demonstrating that this simple electronic transformation can effectively enhance the performance of the optical classifier without adding significant computational overhead. To verify that this procedure learns a meaningful transformation rather than overfitting, we additionally trained **M** on half of the optical outputs and evaluated the classification accuracy on the remaining half, yielding comparable performance as for training on the full output [82% (training) versus 83% (test)].

Intermediate devices between binary and full ten-digit classifiers are presented in Figures S1 (SEMs and height-profile designs) and S5 (confusion matrices from theory and experiment) in the Supporting Information. These results illustrate the progressive scaling of the classification accuracy as the number of encoded classes increases. The all-optical classification accuracies range from 100% for the binary device to 76% for the ten-class implementation, while application of the linear correction matrix **M** improves the accuracies to between 100% and 84%. All experimental results show good agreement (within a few percentage points) with predictions from simple scalar-diffraction theory, as explained in Section S1 of the Supporting Information.

### More Challenging Input Data: Fashion Items

To further test the versatility of our optical-computing concept, we extend it to a more challenging classification task involving items from the fashion MNIST data set.^31^ This data set comprises ten clothing categories such as shoes, T-shirts, and dresses (Figure 3a). In contrast to handwritten digits, these objects exhibit far greater interclass similarity and overlapping spatial features, making them a more demanding benchmark for optical inference. Using the same principles as above, we design and fabricate a diffractive structure with ten sinusoidal gratings encoded within a single reflective silver layer. Again, each grating corresponds to one fashion category and is oriented at a distinct azimuthal angle φ. The resulting device has a maximum depth of 150 nm and a lateral footprint of 40 × 40 μm^2^ (Figure 3b for SEM).

**Figure 3:**
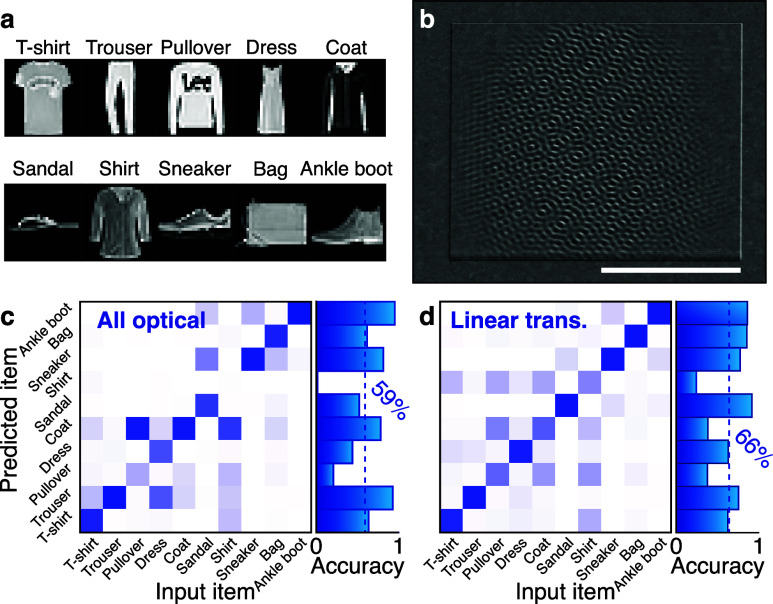
Clothing-item classification. (a) Examples of the 10 classes in the fashion MNIST data set, comprising visually similar clothing categories that present a more challenging classification task than handwritten digits. (b) SEM (30° tilt) of the diffractive silver layer designed for fashion MNIST classification. The scalebar is 20 μm. (c) Confusion matrix for all-optical inference across the 10,000 test images. Inset: single-digit accuracies (histogram) and average accuracy η = 59% (dashed line). (d) Confusion matrix obtained from evaluating all 10,000 fashion MNIST test images using the optically measured outputs combined with the optimized linear calibration matrix **M**. The overall classification accuracy is η = 66%. Misclassification primarily occurs among related categories (e.g., shirts, T-shirts, pullovers, and coats), reflecting intrinsic data set ambiguity.

Evaluation of the device using the complete fashion MNIST test set with 10,000 fashion items yields an all-optical classification accuracy of η = 59% (Figure 3c) and η = 66% when combining the optical outputs with the same linear optimization procedure applied to the digit classifier (Figure 3d). Although this accuracy is lower than for the digit data set, it still exceeds random guessing by more than a factor of 6, demonstrating that substantial feature extraction and class separation occurs in the optical domain. The confusion matrix shows expected difficulties between visually similar categories such as shirts, T-shirts, pullovers, and coats, reflecting the intrinsic ambiguity of the data set rather than device limitations. Intermediate devices with fewer encoded classes, along with their corresponding SEM, height-profile designs, and confusion matrices, are provided in Figures S6 and S7 in the Supporting Information, respectively.

### Broadband Architecture Enables Parallel Computation

To highlight the intrinsic parallelism of our diffractive classifier, we demonstrate that the single-layer digit classifier from Figure 2 can operate at multiple wavelengths in parallel. The key idea is to encode different digit classes not only into distinct azimuthal diffraction angles, as before, but also into different illumination colors spanning the visible spectrum (Figure 4a). Because the classifier diffracts each wavelength into a ring in the back focal plane (Fourier space) with a radius proportional to the normalized in-plane wavevector *k*_∥_/*k*_0_, inputs at different colors naturally separate into concentric circles (Figure 4b). As a result, each wavelength functions as an independent computational channel: a red input encodes one digit and appears at a large diffraction radius, whereas blue inputs map to smaller radii. This wavelength–momentum mapping allows the device to execute many independent classifications simultaneously without crosstalk, limited only by the wavelength spacing Δλ and the numerical aperture (NA) of the collection optics.

**Figure 4:**
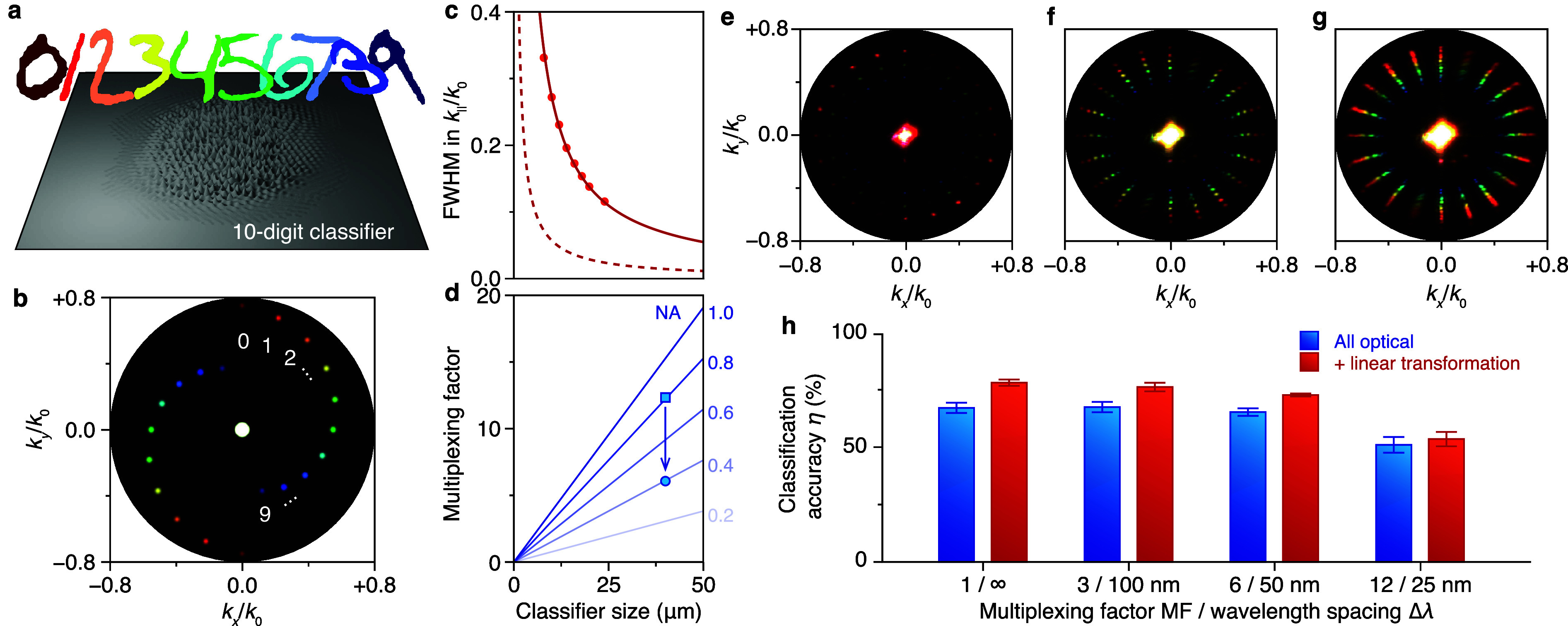
Parallel computations using a broadband digit classifier. (a) Concept of wavelength-multiplexed operation: different digit inputs are encoded at different illumination wavelengths, allowing multiple classifications to be processed in parallel by a single diffractive device. (b) Because the diffraction radius in Fourier space scales with wavelength, different colors map to concentric rings, enabling independent readout channels. (c) Calculated full-width-at-half-maximum (fwhm) of the first-order diffraction peaks in Fourier space as a function of classifier size *L*. The dashed line shows the expected *L* ^–1^ scaling for an ideal sinusoidal grating spanning the full device area. Data points indicate the data set-averaged fwhm obtained by simulating the optical response of 10,000 MNIST training images for different classifier sizes. While the fwhm follows the same *L* ^–1^ scaling (solid line), it exhibits a larger prefactor due to the limited spatial extent of the digit templates within the *L* ^2^ area. (d) Theoretical multiplexing factor, defined as MF = NA/fwhm, indicating the maximum number of resolvable wavelength channels for a given NA as a function of the classifier size. The square indicates the MF value for the present device and collection optics, while the circle accounts for lower effective NA^′^ = 0.4 due to low Ag reflectivity in the ultraviolet. (e–g) Experimentally measured Fourier-space intensities for multiplexing factors of 3 (450, 550, 650 nm), 6 (425–675 nm in 50 nm steps), and 12 (425–700 nm in 25 nm steps), with all 10,000 test inputs superposed. Diffraction orders remain well separated for 3 and 6 channels, while overlap of diffraction orders for different colors becomes apparent for 12 channels. (h) Average classification accuracy (error bars represent standard deviation) as a function of multiplexing factor MF, shown for all-optical inference (blue) and after application of the linear calibration matrix (red). Robust parallel operation is maintained up to six simultaneous wavelength channels, while higher multiplexing leads to performance degradation because of diffraction-order overlap.

To theoretically estimate the limits of wavelength-multiplexed operation, we first analyze the angular width of the diffracted orders in Fourier space as a function of the classifier size. For a single sinusoidal grating that spans the entire device area, scalar diffraction theory predicts a *L* ^–1^ scaling of the spot width (Figure 4c; dashed line), with *L* the side length of the device. However, each handwritten digit occupies only a fraction of the total area *L* ^2^, producing broadened diffraction orders. To capture this effect, we simulate the average Fourier-space response of 10,000 digit-MNIST test images for different *L* and extract the corresponding full-width-at-half-maximum (fwhm; Figure 4c; data points). These data points still follow a *L* ^–1^ scaling, but with a larger prefactor (Figure 4c; solid line). Using this data set-averaged fwhm, we calculate the maximum achievable multiplexing factor (MF) for a given NA, defined as MF = NA/fwhm (Figure 4d), reaching values up to ∼20 under idealized conditions. For our specific device dimensions and collection optics (*L* = 40 μm, NA = 0.8), the accessible multiplexing factor is reduced (Figure 4d, blue square). Additional material constraints further limit the useable spectral range: the reflectivity of silver decreases toward the near-ultraviolet, while the finite numerical aperture restricts the collection of diffraction orders at longer wavelengths as they approach the edge of the collection cone. As a result, only a subset of the angular range can be effectively utilized, corresponding to an effective collection range NA^′^ (Figure 4d, blue circle NA^′^ = 0.4). Consequently, the experimentally accessible multiplexing factor is primarily determined by the device size, collection optics, and available spectral window, with performance ultimately limited by diffraction-order overlap.

We experimentally validate wavelength-multiplexed operation by evaluating the classifier at increasing levels of spectral parallelism, using parallel classification at 3, 6, and 12 different illumination colors (Figure 4e–g). Here, parallel operation is emulated by reconstructing multiwavelength inference in postprocessing from independently measured single-wavelength responses (laser line width of ∼1.5 nm), with inputs randomly selected across digit classes to mimic simultaneous operation. Chromatic aberrations in the imaging optics can introduce slight wavelength-dependent defocus and broadened features in Fourier space; however, by optimizing the collimation for each wavelength, the diffraction spot sizes are minimized and remain limited by the device size rather than by aberrations. The measurements were performed using a silicon-based CMOS detector with high quantum efficiency across the visible spectrum, hence, variations in spectral sensitivity do not affect the multiplexed classification. For 3-fold multiplexing (450, 550, and 650 nm), the diffraction orders of all 10,000 test inputs remain cleanly separated in Fourier space (Figure 4e, all measurements superposed). Increasing to a 6-fold set of wavelengths (425–675 nm in 50 nm steps) still produces clearly distinguishable concentric diffraction rings, consistent with the theoretical multiplexing limit derived above. However, when extending to 12 wavelengths spanning 425–700 nm in 25 nm steps, the diffraction orders overlap. The resulting classification accuracies (Figure 4h) reflect the progressive degradation with increasing spectral crowding: for 3-fold multiplexing the average classification accuracy remains high at 68% all-optical and 76.8% after linear correction; for 6-fold multiplexing we obtain 65.8% and 73.3%; while for 12-fold multiplexing the performance drops to 51.4% and 53.9%, respectively. Notably, when all 12 measurement sets at different wavelengths are evaluated individually, their average classification accuracy (67.7% and 78.5%) remains comparable to the single-wavelength operation in Figure 2, indicating that performance degradation under multiplexed operation arises from diffraction-order overlap rather than a loss of intrinsic device performance for different wavelengths.

The combination of a compact footprint and intrinsic wavelength-multiplexed parallelism enables our diffractive classifiers to reach compute densities, i.e., multiplications per second per square meter, beyond those accessible in digital processors.^32,33^ Each device occupies only 40 × 40 μm^2^, corresponding to a density of 10^10^ m^–2^ computing units that can all process multiple inputs simultaneously through spectral separation. Experimentally, we demonstrate a limit of using 6–12 independent wavelengths, while our theoretical analysis indicates that up to 20 channels are feasible for this architecture. Each optical classification corresponds to a matrix–vector multiplication of a 28^2^-dimensional input onto 10 output channels, amounting to ∼10^4^ physically constrained analog multiplications per inference. When paired with emerging ultrafast optical modulators, such as the LiNbO_3_ spatial-light modulator achieving input rates approaching 10^9^ s^–1^, each optical classifier could perform 10^14^ multiplications per second.^34^ Scaled to a square meter of such devices, this corresponds to a compute density on the order of 10^24^ mathematical operations per second per square meter. Even with conventional commercial SLMs operating at ∼10^2^ Hz, the achievable compute density (10^17^ s^–1^ m^–2^) remains competitive with electronic processors. These estimates highlight the potential of compact diffractive photonic systems to deliver improvements in scalable, low-power, high-throughput computation.

### Digital Benchmarks and Improved Classification

To establish a performance reference for digit classification, we develop a digital model that allows optimization of the input–output transformation. Specifically, we consider a mapping of the form *z* = |**M** *u*|^2^, where **M** is a complex-valued matrix, *u* is the input image, and *z* is the output (Figure 5a). This captures the quadratic nonlinearity introduced by optical detection. As shown in Figure 5b, this model achieves a classification accuracy of 95% on the digit MNIST data set. For comparison, a purely linear model, *z* = **M** *u*, yields 92% accuracy (Figure S8 in the Supporting Information). The gap between the experimental optical performance (76%) and this digital benchmark (95%) primarily reflects the limited expressivity of the analytically constructed diffractive layer rather than a fundamental limitation of the optical approach.

**Figure 5:**
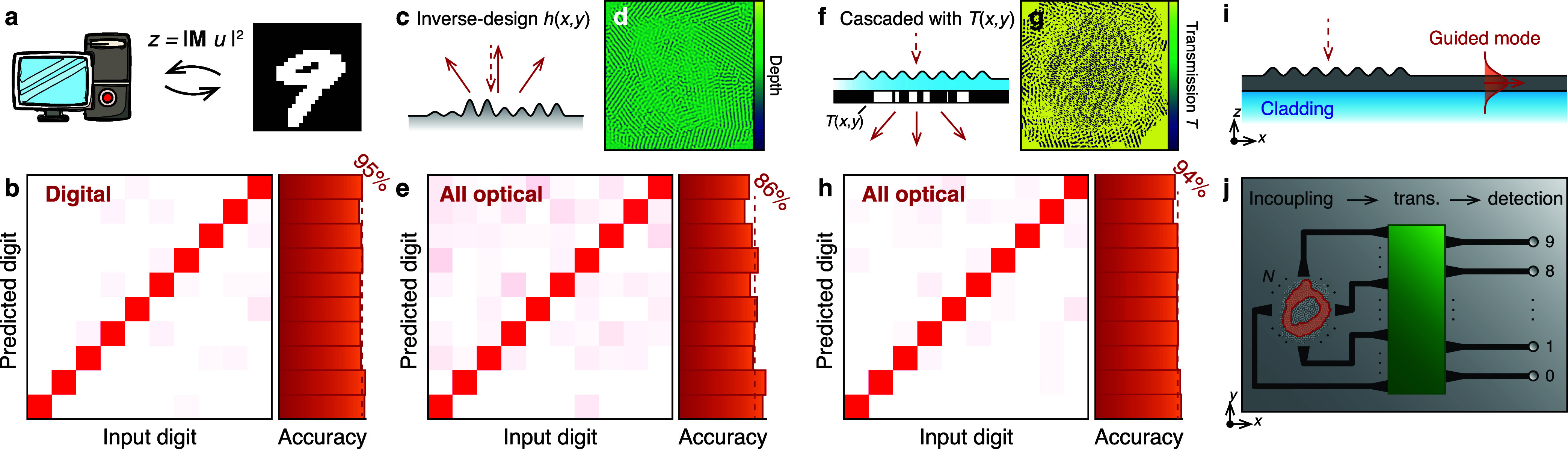
Strategies for improved digit classification. (a,b) Benchmark of an optimized digital classifier using an intensity-based model *z* = |**M** *u*|^2^ between input *u* and output *z*, achieving 95% accuracy. Comparison with a purely linear model *z* = **M** *u* is provided in Figure S8 in the Supporting Information. (c–e) Inverse-designed single-layer reflective processor. (c) Design schematic of a reflective wavy surface with optimizable wavy features, (d) corresponding optimized height profile, and (e) resulting simulated confusion matrix (η = 86%) and single-digit accuracies. (f–h) Cascaded architecture combining the training-free design in transmission with an inverse-designed binary transmission mask. (f) Schematic, (g) optimized transmission mask, and (h) resulting confusion matrix, achieving η = 94% accuracy. (i,j) Concept for hybrid free-space and on-chip photonic processing. (i) Side view (*x,z*-plane) shows coupling of free-space light to a waveguide mode via grating coupling. (j) Top view (*x,y*-plane) illustrating (1) training-free incoupling grating to *N* waveguides, (2) transformation region enabling additional linear and/or nonlinear transformations, and (3) on-chip photodetectors for digit classification.

We theoretically explore how inverse design can improve all-optical digit classification within the same physical framework. Figure 5c–e presents a single-layer reflective processor in which the spatial amplitudes of class-specific wavy features are optimized to maximize classification accuracy. The resulting height profile (Figure 5d) differs from the analytical superposition used in the training-free design (Figure 2a), indicating that the optimal solution requires nontrivial engineering. The inverse-designed processor achieves increased all-optical classification accuracy to η = 86% (Figure 5e). Notably, the training-free implementation already achieved η = 76%, highlighting that the training-free design captured a large fraction of the achievable performance. To improve further, we introduce a cascaded configuration consisting of the training-free processor in transmission combined with an inverse-designed binary transmission mask (Figure 5f,g). This additional degree of freedom results in a much higher classification accuracy of 94% (Figure 5h), even approaching the digital benchmark (Figure 5b).

Finally, further improvements beyond the digital benchmark can be achieved by incorporating nonlinear on-chip processing. One possible implementation is illustrated in Figure 5i,j; where the optical inputs are coupled to waveguide modes on an integrated photonic platform using the training-free processor as a grating coupler (Figure 5j). While such coupling is inherently wavelength- and angle-specific and thus sacrifices the parallelism of the free-space architecture, it enables access to linear and nonlinear transformations on-chip. The coupling process itself provides a structured projection of the free-space field to *N* waveguides, effectively performing preprocessing. Subsequent nonlinear operations, for example, using microring resonators,^18,19^ can then realize more expressive transformations prior to on-chip photodetection. This strategy provides a pathway toward classification performance exceeding that of purely diffractive systems, while maintaining the advantages of training-free optical front-end processing.

## Conclusions

In summary, we have introduced a compact, training-free optical computing platform that performs image classification using a single diffractive layer with a depth variation of only 150 nm. By encoding class-specific spatial-template gratings into a 40 × 40 μm^2^ silver surface, our approach enables all-optical classification with accuracies up to 76% for handwritten digits and 59% for fashion items, which can be further increased to 84% and 66% through a linear transformation of the optical outputs. We demonstrated that the same device could operate as a parallel processor by assigning distinct computational channels to different illumination wavelengths. Theory predicts that up to 20 independent channels can be resolved, and experiments confirm robust performance for multiplexing factors up to six. When combined with emerging ultrafast modulators, these devices can reach compute densities of 10^24^ s^–1^ m^–2^. Finally, inverse-designed architectures suggest that substantially improved all-optical classification (86–94%) is achievable within the same physical framework, with on-chip photonic integration enabling potentially even more expressive optical computing. Our results highlight the potential of compact diffractive photonic architectures for scalable optical computing, where broadband operation and wavelength multiplexing can enable high compute densities.

## Methods

### Fabrication of Optical Processors

The single-layer optical processors were prepared using 1 mm-thick, 2-in.-diameter Si (100) wafers (Silicon Materials). The Si wafers were cleaned using oxygen plasma (GIGAbatch, PVA TePla) at 600 W for 2 min. A 12 wt % solution of poly­(phthalaldehyde) (PPA, Allresist) in anisole (AR 600-02, Allresist) was prepared and 300 μL was spin-coated onto the wafer following a two-step process: first, 5 s of rotation at 500 rpm using a ramp rate of 500 rpm s^–1^; then, 40 s of rotation at 2000 rpm with a ramp rate of 2000 rpm s^–1^. The resulting film was patterned using a NanoFrazer Explore TSPL system (Heidelberg Instruments). For this, custom designs were loaded into the tool, and a depth of 150 nm was chosen. Patterning was performed by scanning a heated cantilever tip over the surface, locally sublimating the PPA. The depth at each pixel was controlled by modulating the electrostatic force between the cantilever and the substrate, allowing precise adjustment of the applied downward force.

An optically thick silver film (>500 nm) was deposited onto the patterned PPA later via thermal evaporation (Nano 36, Kurt J. Lesker). High-purity silver pellets (1/4-in. diameter × 1/4-in. length, 99.999%, Kurt J. Lesker) were used with a deposition rate of 25 Å s^–1^ under a vacuum of approximately 3 × 10^–7^ mbar. Following evaporation, a 1 mm-thick glass microscope slide (Paul Marienfield) was fixed to the silver surface using ultraviolet-curable epoxy (OG142-95, Epoxy Technology). To minimize residual PPA transfer to the final structure, the glass slide was allowed to settle onto the epoxy for ∼5 min prior to curing with ultraviolet light, which was performed afterward for 2 h. Finally, the glass–epoxy–silver stack was mechanically separated from the substrate using a razor blade, revealing the silver surface structure.

### Optical Measurements

The classification measurements were conducted by placing the optical processors on an inverted Nikon microscope (Nikon Eclipse Ti-U) equipped with a 50× air objective (Nikon TU Plan Fluor, NA = 0.8). A schematic of the optical setup is depicted in Figure S2 in the Supporting Information. A supercontinuum laser (SuperK Fianium, NKT Photonics) combined with a tunable band-pass filter (LLTF Contrast, NKT Photonics) provided illumination across a wavelength range of 400–1000 nm. The laser light was guided through an optical fiber (A502-010-110, NKT Photonics), then collimated using a 10× objective (Nikon TU Plan Fluor, NA = 0.3). It then passed through a 750 nm short-pass filter (FESH0750, Thorlabs) to eliminate parasitic light. Subsequently, the light was sent through a broadband 90:10 beam splitter (BSN10R, Thorlabs) to reflect 10% of the light to a power meter. The light from the supercontinuum laser (randomly polarized) was polarized with a linear polarizer (WP25M-VIS, Thorlabs) oriented at 45° relative to the horizontal axis of the SLM to only transmit light with 45° polarization angle. Then, the light went through a visible 50:50 beam splitter cube (BSW10R, Thorlabs) before reaching the reflective SLM­(HOLOEYE PLUTO NIR-11). Amplitude modulation was achieved by placing a second linear polarizer at −45° behind the beam splitter. The amplitude profile was focused on the back focal plane of the 50× air objective by using a defocusing lens with focal distance of 75 cm (ACT508-750 A-ML, Thorlabs) resulting in a demagnification factor of 188. After reflecting off a broadband 50:50 beam splitter (AHF analysentechnik), the amplitude profile was directed to the objective, resulting in a Gaussian illumination profile across the 40 × 40 μm^2^ substrate. The diffracted light from the optical processor, which was positioned at a real plane of the imaging system, was collected in reflection. The reflected light passed through a sequence of lenses (tube lens, Nikon; AC254-200 A-ML, Thorlabs; AC254-200 A-ML, Thorlabs; AC508-200 A-ML, Thorlabs), mirrors, and an iris, before being detected by a digital camera (Zyla PLUS sCMOS, Andor) located in a Fourier plane. The iris, placed in a real plane, was adjusted to collect light exclusively from the classifier.

To ensure optimal performance and reproducibility of the diffraction-based classification, we first verified that the amplitude profile enters the objective at normal incidence by aligning the specular reflection to the center of Fourier space. We optimized the dark–light contrast for the amplitude modulation at various wavelengths by changing the voltage range in the SLM.

We used two standard data sets for machine learning available through the PyTorch module: the digit MNIST (handwritten digits) and fashion MNIST (clothing items) data sets. Both data sets consist of grayscale images of 28 × 28 pixels. The images were binarized by applying a thresholding step: pixels were set to 1 if their intensity exceeded 0.5 for digit MNIST, and 0.1 for fashion MNIST.

### Digital Benchmark with Linear and Quadratic Mapping

Digital models were implemented using optimized mappings of the forms: (1) linear mapping with real-valued **M** (Figure S8), and (2) complex-valued **M** with quadratic mapping (Figure 5a,b). The matrices **M** were trained using gradient descent with a cross-entropy loss on the digit MNIST data set. These models achieve classification accuracies of 92% (linear) and 95% (quadratic), respectively.

### Inverse Design of a Reflective Processor

For the inverse-designed phase plate (Figure 5d), the surface profile was parametrized as a superposition of class-specific sinusoidal gratings (pitch *p* = 1 μm) with trainable spatial amplitudes (50 × 50 grid per grating). The 10 × 50 × 50 amplitudes were optimized using gradient-based learning to maximize classification accuracy on the digit MNIST training set.

### Inverse Design of a Binary Transmission Mask

For the cascaded architecture, the training-free phase plate (Figure 2) was combined with an inverse-designed binary transmission mask (Figure 5g). Optical propagation through the cascaded system was modeled by multiplying the input image with both the transmission mask and phase response, effectively neglecting diffraction between the phase plate and the transmission mask. The mask transmission was optimized to maximize classification accuracy on the digit MNIST training set using gradient-based learning combined with a binarization scheme.

## Supplementary Material



## Notes

Detailed description of the working principle of the optical classifier following scalar diffraction theory (Section S1), and SEMs/designs/confusion matrices of all digit (Figures S1 and S5) and fashion (Figures S6 and S7) classifiers. Optical setup (Figure S2), effect of higher-order diffraction (Figure S3) and lateral misalignment (Figure S4) on classification, and a linear digital benchmark (Figure S8) (PDF)
