# Supplementary material for: Broadband, Compact, and Training-Free Optical Processors for Parallel Image Classification
Source: ACS Nano. 2026 Jun 19;20(26):18719–27. doi: 10.1021/acsnano.6c03355 (PMC13348179; doi:10.1021/acsnano.6c03355)
Supplement: Supplementary file 1 [file nn6c03355_si_001.pdf]

Supporting Information for

Broadband, Compact, and Training-Free Optical Processors for Parallel Image Classification

*Sander J. W. Vonk, Boris de Jong, Yannik M. Glauser, David B. Seda, Matthieu F. Bidaut, Benjamin Savinson, Hannah Niese, and David J. Norris**

Optical Materials Engineering Laboratory, Department of Mechanical and Process Engineering, ETH Zurich, 8092 Zurich, Switzerland

Corresponding Author

*Email: dnorris@ethz.ch

S1. Theoretical Description of Optical Classification by Wavy Classifiers

Light diffraction by wavy optical classifiers can be described using scalar diffraction theory, treating the device as a reflective phase plate because of a spatially varying surface height profile. For a more detailed explanation of wavy diffractive surfaces, we refer to Glauser *et al.*[S1] Here, we consider a real-valued incoming amplitude field $A(x,y)$ (input image) that is reflected by the optical processor, producing a complex-valued electric field in the device plane

$$E(x,y) = A(x,y)\,\exp[2\mathrm{i}\,k_0\,h(x,y)], \tag{S1}$$

where $h(x,y)$ denotes the surface height profile, $k_0 = 2\pi/\lambda$ is the free-space wave number, and λ is the illumination wavelength. The factor of 2 accounts for the reflective geometry, for which the optical path difference is twice the surface height. This electric-field profile propagates away from the device and redistributes the optical energy through diffraction. In the far field (or in Fourier space that we measure in the back focal plane of our microscope objective), we simply find the intensity profile I_{out} through the two-dimensional Fourier transform $\mathcal{F}\{\cdot\}$ of the electric field in the device plane, via

$$I_{\mathrm{out}} \propto |\mathcal{F}\{E\}|^2. \tag{S2}$$

The working principle of optical classification follows from this Fourier-space representation. Each optical classifier consists of a superposition of sinusoidal gratings with a period $p = 1$ µm, where each grating is spatially weighted by the average spatial distribution of a specific class. Upon illumination, spatial overlap between the input image $A(x,y)$ and the corresponding class-specific grating template boosts the ± 1 diffraction orders associated with that grating. For a sinusoidal grating of period p oriented at an azimuthal angle φ, the ± 1 diffraction orders appear at well-defined in-plane wavevectors $\mathbf{k}_{\parallel} = (k_x, k_y)$ given by

$$\frac{\mathbf{k}_{\parallel}}{k_0} = \pm\frac{\lambda}{p}\begin{pmatrix}\cos\varphi\\ \sin\varphi\end{pmatrix}. \tag{S3}$$

All single-color outputs lie on a circle of radius $k_{\parallel}/k_0 = \lambda/p$ in Fourier space, with their radial positions determined by the illumination wavelength. These mappings in eq S3 show both

the azimuthal angular separation of output channels and the wavelength-multiplexed operation discussed in Figure 4 of the main text.

To simulate the optical response and evaluate classification performance, we numerically compute eq S2 using class-specific illumination patterns $A(x, y)$ derived from the digit MNIST and fashion MNIST datasets. For each input image, the resulting Fourier-space intensity distribution is analyzed to identify the pairs of angularly separated ±1 diffraction orders corresponding to the different output ports. Classification is performed by assigning each input to the class associated with the diffraction channel exhibiting the highest integrated intensity, following the procedure described in eq 2 of the main text. Simulated classification results for digit and fashion item classifiers are summarized as confusion matrices and single-class accuracy histograms in Figures S5 and S7, respectively.

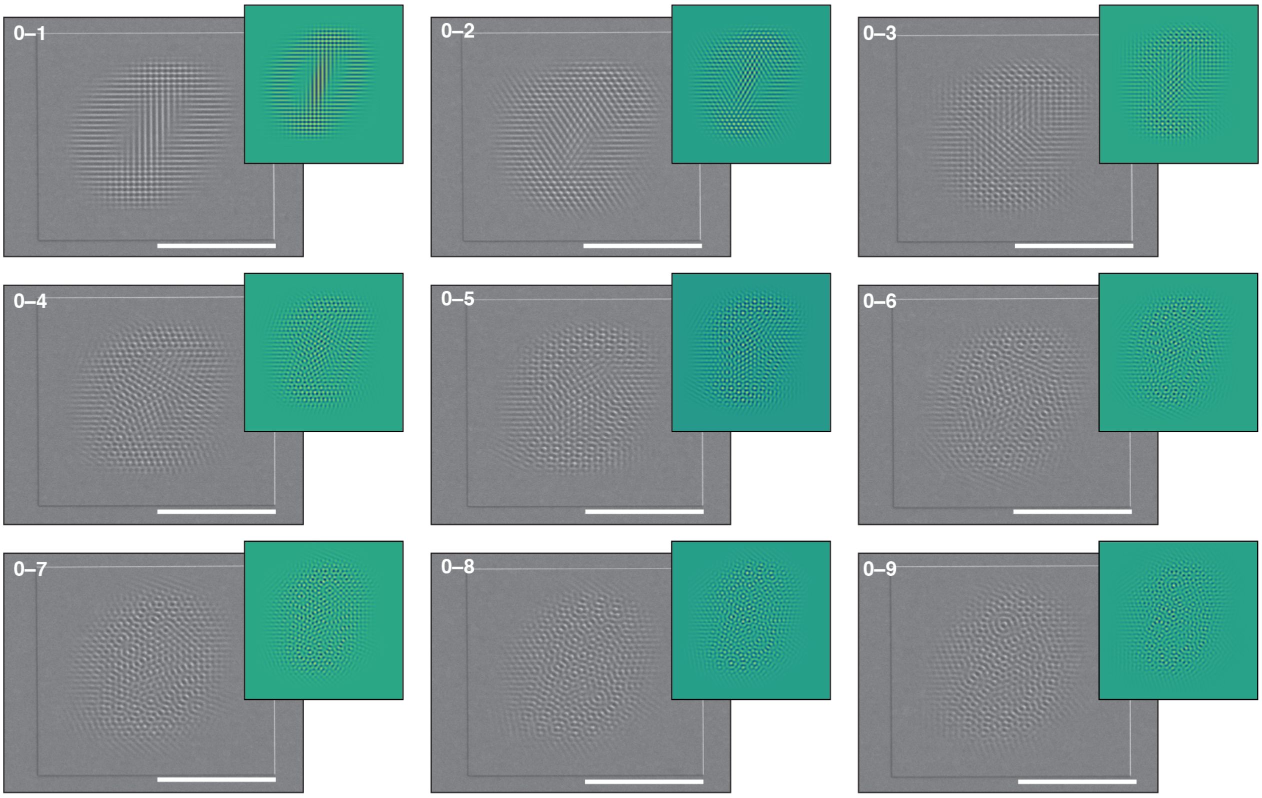

Figure S1. Scanning electron micrographs and designs of digit classifiers. Scanning electron micrographs (SEMs) of all digit classifiers, where the indicated digit range (top left, e.g., 0–9) denotes the set of digit classes contained in each classifier. The number of digit classes was systematically increased, ranging from a 0–1 classifier to a 0–9 classifier. The classifier designs (height profiles) are shown as insets. The scanning electron microscope was operated at 10 kV, and the images were collected at a 30° tilt. All scale bars are 20 μm.

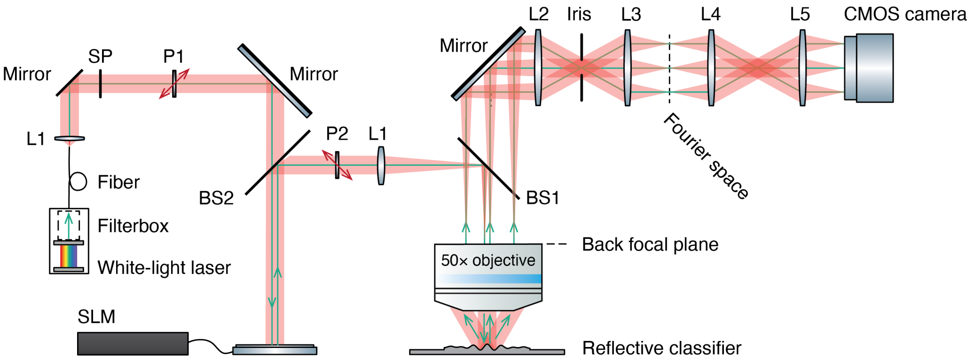

Figure S2. Optical setup for image classification. A supercontinuum white-light laser (SuperK Fianium supercontinuum laser, NKT Photonics) combined with a tunable filterbox provides illumination in the 400–1000 nm range. The beam is fiber-coupled, collimated (L1), and spectrally cleaned using a 750-nm short-pass (SP) filter. Amplitude control is achieved using linear polarizers oriented at ±45° (P1 and P2) relative to the spatial light modulator (SLM). The modulated beam is shaped and relayed through a defocusing lens to the back focal plane of a 50× objective, producing a Gaussian illumination profile over the 40×40 μm² sample area. The reflective classifier is positioned in the image plane, and the diffracted light is collected via the same objective. The signal is relayed through a series of tube (L2), relay (L3 and L5), and Fourier (L4) lenses, mirrors, and an iris aperture (spatial filtering in a real plane) before detection by a CMOS camera placed in a Fourier plane. The iris ensures that only light originating from the classifier contributes to the recorded signal.

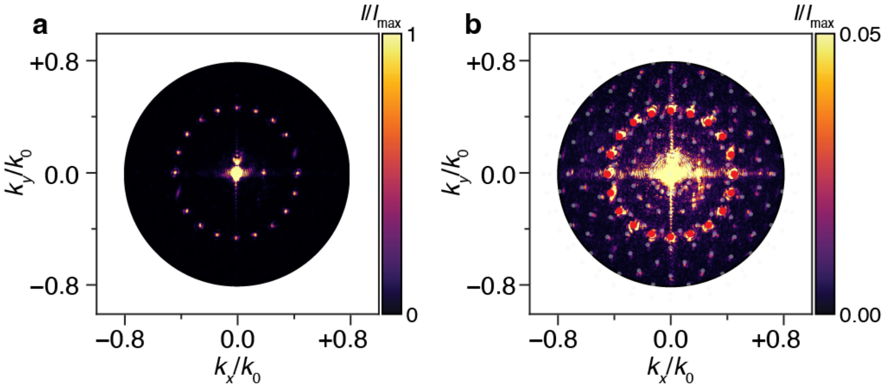

Figure S3. Higher-order diffraction. (a) Measured optical response of the full-digit classifier (Figure 2 in the main text), summed over all 10,000 input digits of the MNIST test set. The 20 diffraction spots at $|k_{||}\ /k_0| = 0.45$ arise from first-order diffraction of 450-nm illumination. The saturated bright spot at $|k_{||}/k_0| = 0$ corresponds to specular reflection of the incident light field coming from the surface normal. (b) Same measurement as in (a), with the intensity scale clipped to 5% of the maximum intensity. Additional diffraction orders appear next to the first-order diffraction peaks (red dots). These features are attributed to second-order diffraction arising from different combinations of the 10 grating directions, for which the transparent dots indicate the expected diffraction directions. Because the amplitude of each sinusoidal grating is much smaller than the wavelength, second-order diffraction is significantly less efficient than first-order diffraction and therefore has a negligible effect on image classification.

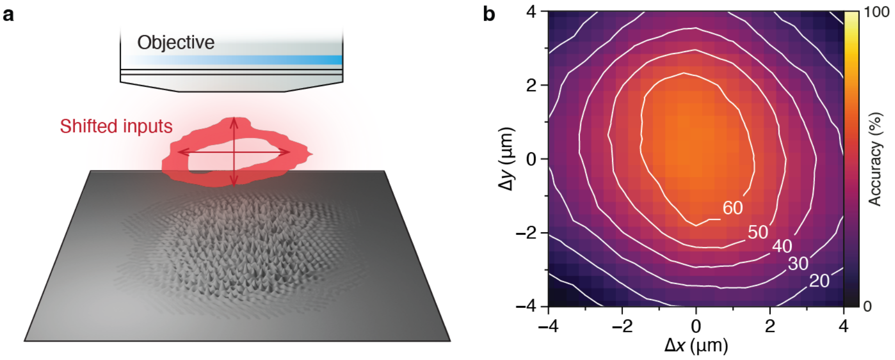

Figure S4. Influence of lateral input misalignment on classification accuracy. (a) Schematic illustration of the problem, showing lateral displacements of the optical input relative to the reflective diffractive classifier. (b) Simulated classification accuracy as a function of lateral displacement Δx and Δy, evaluated over the range ± 4 μm. The color map represents the all-optical classification accuracy, with iso-accuracy contour lines (white) overlaid. The maximum accuracy reaches 69.2% at optimal alignment and decreases with increasing displacement. Accuracy drops below 60% for $|\Delta x| > 1$ μm and $|\Delta y| > 2$ μm, indicating a stronger sensitivity to horizontal misalignment. Simulations were performed using 100 test images per digit class.

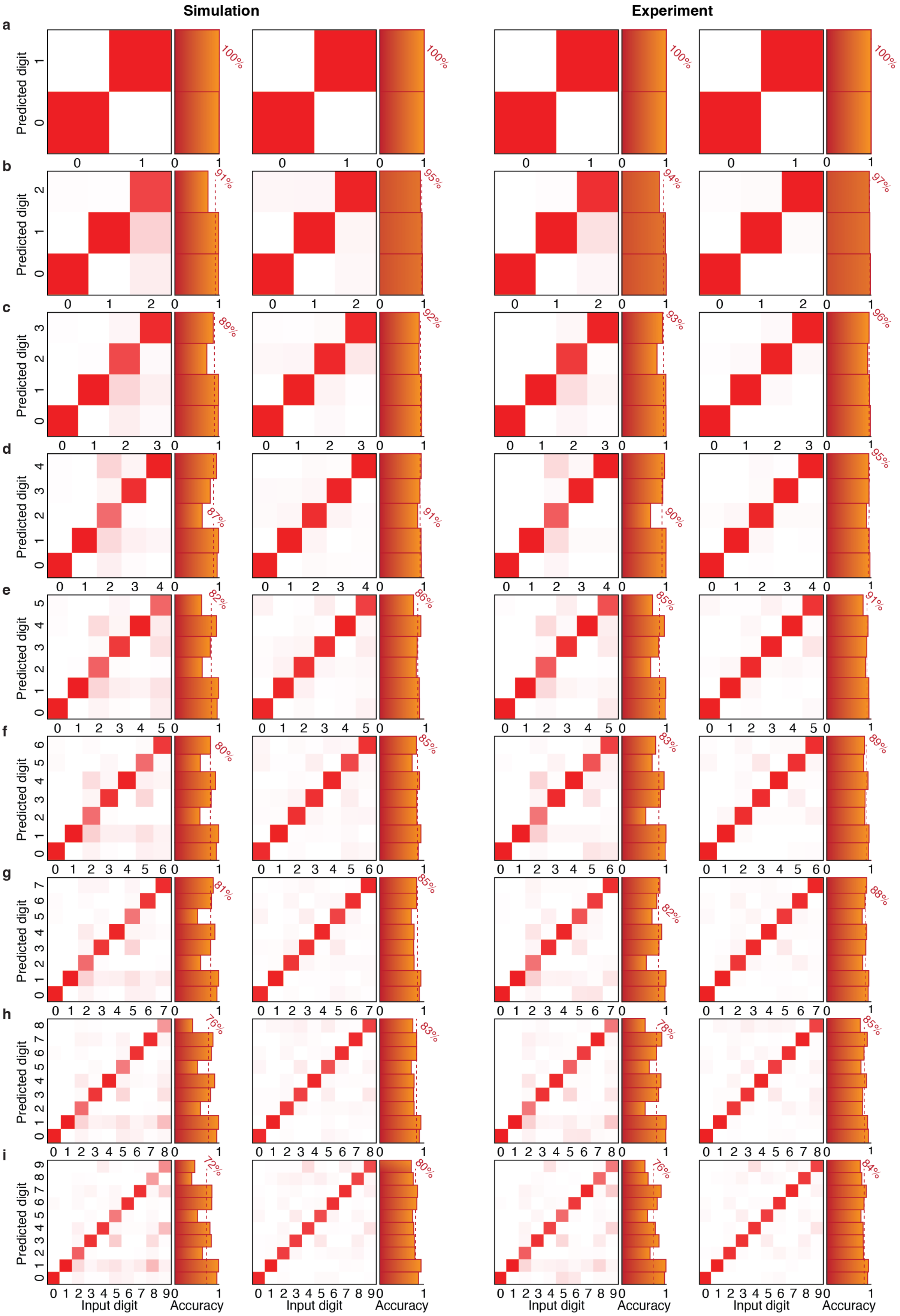

Figure S5. Digit classification accuracy. (a) Confusion matrices for binary 0–1 classification: the two left panels are obtained directly from optical outputs (simulated by scalar diffraction theory or measured experimentally), while the two right panels show results after linear transformation of the optical data using an optimized matrix $\mathbf{M}$. (b–i), Same as (a), but for digit classification from 0–2 up to full 0–9 classification.

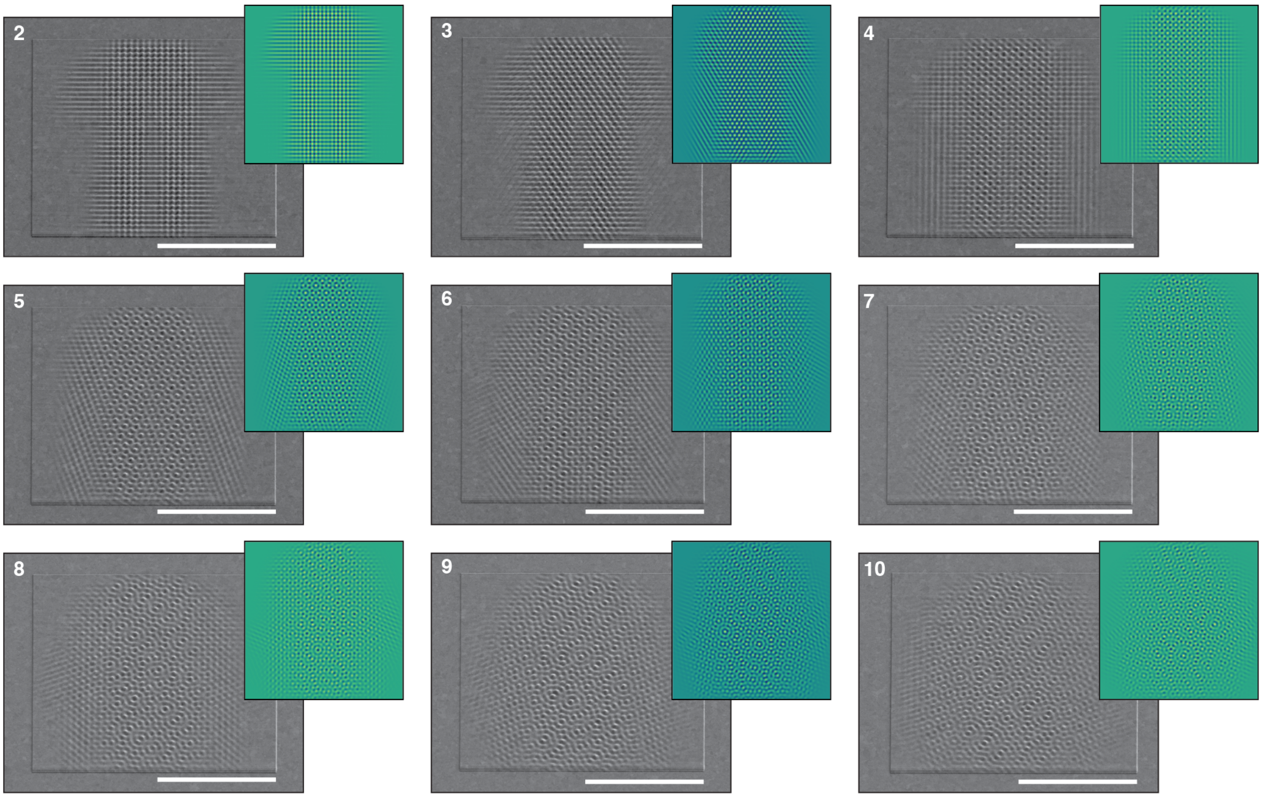

Figure S6. Scanning electron micrographs and designs of fashion-item classifiers. SEMs of all fashion item classifiers, where the indicated digit (top left) denotes the number of fashion classes contained in each classifier. The classifiers always include ‘T-shirt’ as the first class and progressively incorporate additional classes until all fashion MNIST classes, including ‘ankle boot’ as the final class, are contained. The classifier designs (height profiles) are shown as insets. The scanning electron microscope was operated at 10 kV, and the images were collected at a 30° tilt. All scale bars are 20 μm.

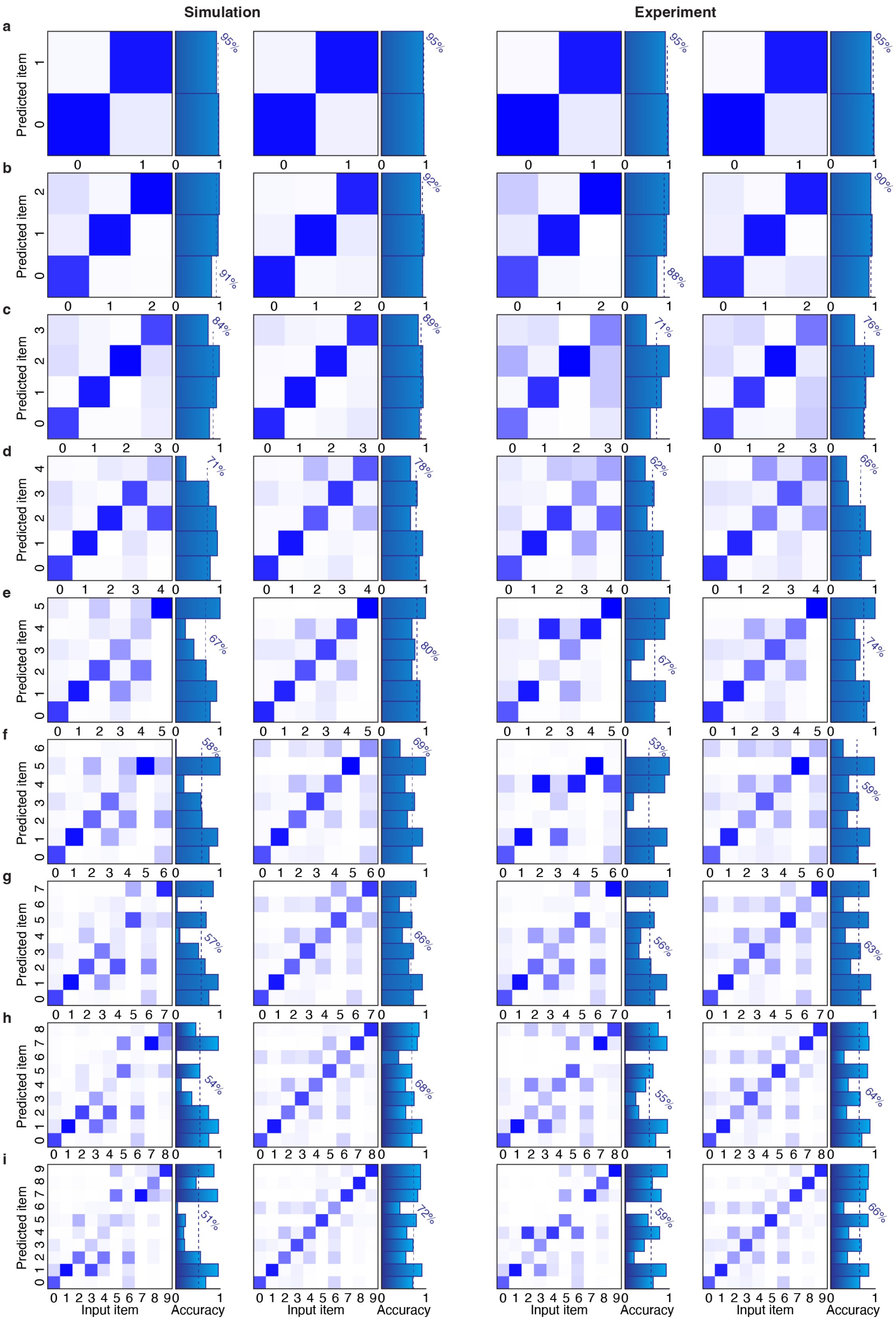

Figure S7. Fashion item classification accuracy. (a) Confusion matrices for binary T-shirt–trouser classification: the two left panels are obtained directly from optical outputs (simulated by scalar diffraction theory or measured experimentally), while the two right panels show results after linear transformation of the optical data using an optimized matrix $\mathbf{M}$. Fashion-item classes are depicted by a number between 0 (T-shirt) and 9 (ankle boot). (b–i) Same as (a), but for fashion-item classification for 3 up to full 10-class classification.

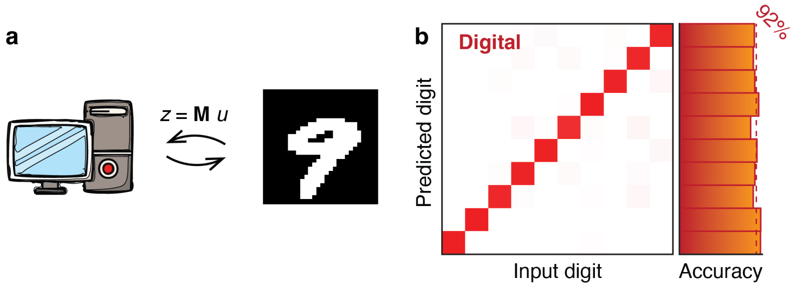

Figure S8. Digital linear classification. (a) Schematic illustration of the linear classification model $z = \mathbf{M}\,u$, where the input image u is transformed by an optimized matrix $\mathbf{M}$ to produce outputs z. (b) Corresponding confusion matrix and single-digit classification accuracies obtained on the digit MNIST test set, yielding an average accuracy of $\eta = 92\%$.

Supporting References

(S1) Glauser, Y. M.; Maris, J. J. E.; Brechbühler, R.; Crimmann, J. G.; De Rosa, V. G.; Petter, D.; Nagamine, G.; Lassaline, N.; Norris, D. J. Diffraction of Light from Optical Fourier Surfaces. *ACS Photonics* **2025**, *12*, 2664–2675.
